# Methamphetamine use is associated with high levels of depressive symptoms in adolescents and young adults in Rural Chiang Mai Province, Thailand

**DOI:** 10.1186/s12889-016-2851-1

**Published:** 2016-02-19

**Authors:** Lauren E. DiMiceli, Susan G. Sherman, Apinun Aramrattana, Bangorn Sirirojn, David D. Celentano

**Affiliations:** Department of Epidemiology, Johns Hopkins Bloomberg School of Public Health, 615 N. Wolfe Street, Baltimore, MD 21205 USA; Research Institute for Health Sciences, Chiang Mai University, Chiang Mai, Thailand

**Keywords:** Thailand, Methamphetamine, Depressive symptoms, Epidemiology, Adolescence, Substance abuse

## Abstract

**Background:**

High levels of depressive symptoms often occur among individuals that use or that are dependent on methamphetamine (MA). Thailand is currently experiencing an epidemic of MA use among youth. Understanding the nature of the relationship between depressive symptoms and MA use and identifying those most at risk can further understanding of prevention and treatment options for youth who use MA and present with depressive symptoms.

**Methods:**

In 2011, we conducted a cross sectional epidemiologic study that examined associations between MA use and high levels of depressive symptoms among adolescents and young adults aged 14–29 living in Chiang Mai province, Thailand. A combination of cluster and systematic sampling was conducted to obtain a study sample of participants actively recruited in Chiang Mai province. Depressive symptoms were measured using a Thai translation of the Centers for Epidemiologic Studies Depression scale (CES-D). The independent variables measured reported lifetime and recent MA use within the past 3 months. Multivariate logistic regression models were used to assess associations between MA use and high levels of depressive symptoms.

**Results:**

Approximately 19 % (*n* = 394) of the sample reported ever having consumed MA and 31 % (*n* = 124) of lifetime users reported recent MA use within the past 3 months. Recent MA use was associated with high levels of depressive symptoms (aPOR recent use: 2.60, 95 % CI: 1.20, 5.63).

**Conclusions:**

This is one of the first studies to examine the association between MA use and high levels of depressive symptoms in a general Thai population. The odds of having high levels of depressive symptoms was significantly greater among recent MA users compared to non-users. These findings support the need for policies, programs and interventions to prevent and treat depressive symptoms presenting among MA using Thai adolescents and young adults in rural Chiang Mai province, Thailand to aid in cessation of MA use. Furthermore, additional research is needed to investigate treatment options for adolescents and young adults in Thailand that use MA and present with high levels of depressive symptoms.

## Key messages

- This research demonstrates an association between methamphetamine and depressive symptoms, controlling for confounding, among a general population of Thai adolescents and young adults in Chiang Mai Province, Thailand.
- This research provides evidence to support the need for interventions to target this comorbidity among young people in rural areas of Thailand that are exposed to methamphetamine and serves as a foundation for further research into effective treatment of depressive symptoms and cessation of methamphetamine use among youth in rural Northern Thailand.

## Background

Methamphetamine (MA) use became ubiquitous as a recreational drug among adolescents and young adults in Thailand beginning in the mid-1990s [1, 2]. The popularity of recreational MA use among Thai adolescents and young adults, the perceptions about MA use being normative or fashionable, exposure to MA from peers, personality traits such as curiosity and sensation-seeking, coping mechanisms and functional needs led to the emergence of MA as a significant public health problem among this population [3, 4]. Indeed, MA use has a lasting psychosocial impact not only on individuals, but entire communities [5]. MA became commonly referred by Thais as “ya-ba,” or crazy pill, due to its ability to induce psychosis and other psychological symptoms [6]. By 2003, an estimated 3 million Thais or 5 % of the population were reported to be chronically dependent on MA [7].

MA use has been shown to be associated with severe psychological harms and commonly is associated with depression [8]. Studies in various settings have found a high prevalence of MA users reporting a lifetime history of depression, with prevalence estimates ranging between 50 % of men and 68 % of women and 57 % of MA users reporting the presence of depressive symptoms in the past year compared to 32 % among non-users [9, 10]. Another study conducted among MA users entering treatment in Australia observed that 40 % (*n* = 400) of study participants had a diagnosis of major depression within a year prior to admission, and another 44 % met the criteria for major depression but were excluded because their depressive symptoms were attributed to drug dependence [11]. In a study of young Thai MA users aged 18–25 years, authors observed a prevalence of 35 % of participants presenting with high levels of depressive symptoms [12]. Most studies are limited by design or methodology because they have assessed the prevalence of depression among amphetamine or MA users using various instruments to measure depressive symptoms, and most have examined limited data among health seeking populations that may not be representative of all users. Most of these studies have not been conducted among populations in low- and middle-income countries and only one was conducted among Thai youth. No studies of general Thai populations in rural areas have been conducted to our knowledge.

The nature of the relationship between depressive symptoms and MA use, abuse and dependence (i.e., drug-induced depressive symptoms, self-medicating to alleviate depressive symptoms, etc.) and the prevalence of these comorbid conditions have public health implications for treatment and prevention that have received little attention outside of the United States. Thailand, which is currently experiencing an MA epidemic, warrants further attention in terms of prevention and treatment of these comorbid conditions [12]. Populations residing in Thailand’s Northern provinces, including Chiang Mai province, are the most accessible to MA trafficking routes since the drug is distributed across the borders separating Thailand from Laos and Burma, and many residents are involved in the drug trade [7]. High levels of depressive symptoms may be greater than average among Thai adolescents and young adults in these rural areas within the Golden Triangle due to exposure to and participation in the MA market and the extensive use of MA within social networks. However, research has yielded little documentation of high levels of depressive symptoms comorbid to MA use in Thailand and little is known of evidenced-based or culturally-relevant interventions to prevent and treat both disorders. Furthermore, mental health and substance abuse research have received little attention in Thailand compared with the magnitude of publications dedicated to medical research [13].

Our research attempts to add to the growing body of knowledge of mental health and substance use research emerging in Thailand using probabilistic sampling methods that improve the generalizability of results to the general population of adolescents and young adults in rural areas of northern Thailand where MA distribution and use is prominent. The primary purpose of this study is to describe the prevalence of depressive symptoms among MA users and to assess the association between MA use and depressive symptoms among Thais 14 – 29 years of age residing in rural areas of northern Thailand in close proximity to the Burma and Lao borders. We hypothesize that there will be an association between MA use and high levels of depressive symptoms.

## Methods

### Study population

This study uses data from a baseline survey of behavioral data administered prior to the start of a community cluster-randomized community mobilization trial in six districts of rural Chiang Mai province in northern Thailand. Baseline data collection took place within a complex combination of nested cluster and systematic random sampling, beginning at the district level and proceeding with cluster sampling of sub-districts, communities and villages. Next, systematic random sampling was conducted to sample from a frame consisting of households and finally individuals, which yielded a representative sample of adolescents and young adults aged 14–29 years of age. The response rate during recruitment procedures was over 90 % (*n* = 2055). The study has been described in detail elsewhere [14]. This research was approved by the Institutional Review Board (IRB) at the Johns Hopkins Bloomberg School of Public Health, the Human Experimentation Committee at the Research Institute for Health Sciences, Chiang Mai University and the Faculty of Medicine, Research Ethics Committee, Chiang Mai University, Thailand. Written informed consent for participation in this study was obtained from participants or, if participants were adolescents 14–17 years of age, informed assent and informed consent from a parent or guardian was obtained.

### Case definition

Depressive symptoms were measured by using the Center for Epidemiologic Studies Depression Scale (CES-D) [15]. The CES-D contains 20 items that measure self-reported symptoms of depression experienced for a duration of at least 2 weeks. Participants report the frequency of experiencing each item on a scale ranging from 0, indicating that they experience that symptom rarely or none of the time, and 3, indicating that they experience that symptom most or all of the time. Items are summarized during the statistical analysis to obtain a score capable of ranging from 0 to 60 and scores of the four positive items were reversed prior to generating total scores. A cutoff of  ≥ 22 was used to define high levels of depressive symptoms among Thais because this cutoff has been previously validated in Thailand [16]. This cutoff was based on an evaluation of the CES-D against structured interviews, conducted by psychiatrists, of adolescent Thai males aged 15–18; participants with validated depression, based on interviews with psychiatrists, had significantly higher CES-D scores when compared to participants without depression. The study determined a cutoff of  ≥ 22 produced optimal sensitivity and specificity (sensitivity = 72 %, specificity = 85 %). To our knowledge, no other scale used to measure depressive symptoms has been validated among a general Thai population of adolescents and young adults with ages similar to the population in this study [12, 16, 17].

### Variables

Items used to measure drug use behaviors were obtained from portions of the Risk Behavior Assessment (RBA) questionnaire [18]. MA use patterns were defined as lifetime use if the participant reported ever using MA and defined as recent use if the participant reported using MA in the past 3 months. Responses derived from these two questions were coded as binary variables whereby 0 indicated no use and 1 indicated use of MA on at least 1 day during their lifetime or within the past 3 months. Confounders identified from the literature were measured and included age, gender, educational attainment, lifetime alcohol consumption, recent alcohol consumption and the use of illicit drugs other than MA within the participant’s lifetime or in the past 3 months prior to baseline [12]. To measure lifetime and recent alcohol consumption within the past 30 days, we used portions of the Alcohol Use Disorders Identification Test (AUDIT) [19]. Illicit drugs included: ice, heroin, opium, valium, domicum, barbiturates, marijuana, ketamine, glue and kratom, another illicit drug commonly used in Thailand [20]. Responses on reported illicit drug use were summed and coded as a binary variable similar to responses obtained from questions about MA use. Age was grouped into two categories defined by adolescents being 14–17 years of age and young adults being those 18–29 years of age; young adults were thought to be at different developmental stages compared to adolescents. Adolescents may vary in expression of depressive symptoms and endorsement of certain items on the CES-D when compared to young adults [21]. Measures of educational attainment included reports of never having gone to school or the participant’s self-report of having completed primary school, secondary school, high school, vocational school or of having earned a college degree or more. Additional measures of education included items to assess whether the participant was currently attending school.

### Statistical analysis

Comparisons were made between sociodemographic and substance use variables between those categorized as having high levels of depressive symptoms and those having low levels of depressive symptoms using chi-squared tests. Crude logistic regression was implemented to examine associations between lifetime and recent MA use and high levels of depressive symptoms. Crude logistic regression models were also conducted to assess associations between sociodemographic or drug use variables and high levels of depressive symptoms to assess potential confounders. Covariates that achieved a level of statistical significance of *p* <0.10 and variables that have been identified in the literature as confounders were included in the final multivariate logistic regression model. Because the data were cross-sectional, prevalence odds ratios (POR) with 95 % confidence intervals were estimated to compare the odds of having high levels of depressive symptoms between reported MA users against a reference group of abstainers. Due to clustering of individuals by district, a sensitivity analysis was performed to account for the clustered nature of the data by incorporating the “xtlogit” command in Stata as an extension of multivariate logistic regression models [22]. Interactions by gender and use of illicit drugs other than MA were incorporated into models to assess effect modification. The prevalence of MA use and of high levels of depressive symptoms among lifetime and recent MA users was also determined. Statistical analyses were conducted using Stata Intercooled version 10.1 (Stata Corp., College Station, Texas).

## Results

Descriptive statistics can be found in Table 1. The sample (*n* = 2055) consisted predominately of young adults (*n* = 1247) with a median age of 20 who were predominantly single, separated, widowed or divorced (79 %), of Thai ethnicity (100 %), and Buddhist (99 %) and reported living primarily at their parent’s house during the past 3 months (77 %), currently attending school (59 %), and currently being unemployed (56 %). Approximately 19 % reported ever having consumed MA (*n* = 394) with 31 % of lifetime users reported recent MA use within the past 3 months (*n* = 124). Relative to those without reported baseline lifetime MA use, individuals reporting ever having consumed MA were significantly more likely to be male (*p* < 0.001).

**Table 1:** Characteristics of the Sample and CES-D Categories

Baseline Characteristics	High Levels of Depressive Symptoms	Low Levels of Depressive Symptoms	All	*p*-value
	*N* = 182	*N* = 1873	*N* = 2,055	
	*N* (%)	*N* (%)	*N* (%)	
Gender				
Males	64 (35.2 %)	987 (52.7 %)	1051 (51.1 %)	<0.05
Age				
14–17	58 (31.9 %)	750 (40.0 %)	808 (39.3 %)	
18–29	124 (68.1 %)	1123 (60.0 %)	1247 (60.7 %)	<0.05
Residence				
Parent’s house	145 (79.7 %)	1441 (76.9 %)	1586 (77.2 %)	
Other	37 (20.3 %)	432 (23.1 %)	469 (22.8 %)	0.401
Marital status				
Single, widowed, separated, divorced	133 (73.1 %)	1491 (79.6 %)	1624 (79.0 %)	
Currently married or cohabitating	49 (26.9 %)	382 (20.4 %)	431 (21.0 %)	<0.05
Religion				
Buddhism	181 (99.5 %)	1850 (98.8 %)	2031 (98.8 %)	____
Ethnicity				
Thai	182 (100 %)	1871 (99.9 %)	2053 (99.9 %)	____
Current school attendance				
Yes	89 (48.9 %)	1123 (60.0 %)	1212 (59 %)^a^	<0.05
Employment status				
Full-time, part-time, irregular work	88 (48.4 %)	738 (41.0 %)	817 (41.4 %)^b^	0.236
MA Use				
Lifetime use	46 (25.3 %)	348 (18.6 %)	394 (19.2 %)	<0.05
Recent use	20 (3.5 %)	104 (29.9 %)	124 (31.5 %)^c^	<0.10
Illicit Substances				
Lifetime use	41 (22.5 %)	341 (18.2 %)	382 (18.5 %)	0.15
Recent Use	5 (12.2 %)	74 (21.7 %)	79 (20.7 %)^d^	0.16
Alcohol Consumption				
Lifetime use	131 (72.0 %)	1370 (73.1 %)	1501 (73.0 %)	0.74
Recent use	85 (64.9 %)^e^	858 (62.6 %)	943 (62.8 %)^e^	0.61

Table 1 describes the distributions of the general characteristics of the total sample and two subpopulations of the sample defined by high and low depressive symptoms based on the recommended. Thai cutoff of ≥ 22. Recent MA and polydrug use is defined as the proportion of lifetime users reporting recent substance use. Illicit substances is defined as all other illicit substances other than MA and includes use of one or more of the following: heroin, opium, valium, domicum, barbiturates, marijuana, ketamine, glue and kratom

*p*-values indicate chi-squared test for independence between high/low levels of depressive symptoms and sample characteristics. Frequencies and proportions of study participants reporting recent drug or alcohol use are subsets of participants who reported lifetime use

^a^ *n* = 2054

^b^ *n* = 1972

^c^ *n* = 394

^d^ *n* = 382

^e^ *n* = 1501

Approximately 9 % of the sample (*N* = 2055) met criteria for exhibiting high levels of depressive symptoms. The mean CES-D score estimated for the entire sample was 17.01 (S.D.: 6.39). The range of CES-D scores was 0–57. Among lifetime MA users, the prevalence of high levels of depressive symptoms was approximately 12 % (*n* = 46) compared to 8 % of abstainers and 16 % among recent MA users compared to 10 % of abstainers. Relative to those who reported no lifetime MA use at baseline (Table 2), individuals who reported having ever used MA in their lifetime were more likely to have high levels of depressive symptoms using the recommended Thai cutoff score of ≥ 22 on the CES-D scale, but this measure of association did not reach statistical significance (POR: 1.48, CI: 1.04–2.11; *p* <0.05). Of those who reported a history of lifetime MA use, recent MA users were almost twice as likely to have high levels of depressive symptoms compared to nonusers (POR: 1.80, CI: 0.96–3.38; *ρ* <0.10). Compared to those without high levels of depressive symptoms, those defined as having high levels of depressive symptoms at baseline were more likely to be female (*p* < 0.001). Table 1 also describes the distributions of high levels of depressive symptoms among various subgroups of the sample. Additionally, the proportion of female MA users having high levels of depressive symptoms was substantially higher than male MA users, with 23 % of females who reported ever using MA experiencing high levels of depressive symptoms compared to 9 % of males (*p* < 0.01).

**Table 2:** Crude Prevalence Odds Ratios Indicating Odds of Endorsing High Levels of Depressive Symptoms among Demographic and Drug Use Behavior Characteristics

	Crude prevalence odds Ratio	Confidence Interval	*ρ*-value
Age	1.43	1.03–1.98	<0.05
Gender	2.05	1.50–2.82	<0.001
Highest level of education^a^	0.93	0.66–1.33	0.71
Current school attendance	1.57	1.15–2.13	<0.05
Employed^b^	1.21	0.88–1.65	0.24
Residence	0.93	0.66–1.33	0.71
Lifetime MA use	1.48	1.04–2.11	<0.05
Recent MA use^c^	1.80	0.96–3.38	<0.10
Lifetime polydrug use	1.31	0.91–1.89	0.15
Recent polydrug use^d^	0.50	0.19–1.32	0.16
Lifetime alcohol consumption	0.94	0.67–1.32	0.74
Recent Alcohol consumption^e^	1.10	0.76–1.60	0.61

Age categorized into two groups: 14–17 years of age and 18–29 years of age

Employed categorized into Full-time, part-time or irregular work compared to unemployed. Highest level of education categorized primary, secondary or high school compared to vocational school or college. Residence categorized as living with parents compared to living without parents

*MA* methamphetamine

^a^ *N* = 2046

^b^ *N* = 1972

^c^ *N* = 394

^d^ *N* = 382

^e^ *N* = 1501

We examined demographic and behavioral variables identified in the literature as confounders. In our study, associations between high levels of depressive symptoms and age, gender and current school attendance were statistically significant (Table 2). In other words, the odds of having a high level of depressive symptoms was greater among females, young adults, individuals not currently attending school, and MA users. Due to concerns about collinearity, we used current school attendance in the final model rather than educational attainment. Multivariate logistic regression (Table 3) demonstrated significant, independent associations between recent MA use and high levels of depressive symptoms (POR: 2.60, 95 % CI: 1.20, 5.63). Statistical models which accounted for clustering by using the “xtlogit” command in Stata generated similar prevalence odds ratios (data not shown) and there was no evidence for effect modification of high levels of depressive symptoms by MA use and polydrug use or by MA use and gender.

**Table 3:** Adjusted prevalence ratios derived from logistic regression for high levels of depressive symptoms, MA use and covariates to describe odds of high levels of depressive symptoms among adolescents and young adults who use MA in northern rural Thailand

	Adjusted prevalence odds ratio^a^	Confidence intervals	*Ρ*-value
Lifetime MA Risk Behavior (*N* = 2054)			
Lifetime MA use	1.61	0.98–2.64	0.06
Lifetime polydrug use *N* = 2054	1.45	0.87–2.43	0.16
Lifetime alcohol consumption	0.82	0.55–1.23	0.33
Gender	2.63	1.82–3.80	<0.001
Age	1.16	0.75–1.77	0.49
Current school attendance	1.35	0.92–1.99	0.13
Recent MA Risk Behavior (*n* = 266)			
Recent MA use	2.60	1.20–5.63	<0.05
Recent polydrug use	0.53	0.17–1.67	0.23
Recent alcohol consumption	0.85	0.26–2.75	0.79
Gender	3.31	1.28–8.55	<0.05
Age	0.93	0.30–2.87	0.90
Current school attendance	1.19	0.43–3.33	0.74

CES-D denotes the Center for Epidemiologic Studies Depression Scale (Radloff [15])

^a^Adjusted for gender, age, current school attendance, lifetime or recent polydrug use of any illicit drug other than MA use within the past 3 months, and lifetime or recent alcohol consumption of any beverage within the past 30 days. Reported measures of lifetime alcohol and polydrug use are included in regression models assessing the relationship between lifetime MA use and high levels of depressive symptoms. Recent alcohol consumption and polydrug use are included in regression models assessing the association between recent MA use and high levels of depressive symptoms. Polydrug use defined as reported use of one or more of the following: ice, heroin, opium, valium, domicum, barbiturates, marijuana, ketamine, glue and kratom

## Discussion

High levels of depressive symptoms were associated with reported recent MA use. This study quantifies the prevalence of high levels of depressive symptoms comparing MA users to non-MA users in a general population of adolescent and young Thai adults. The study also examines the association between depressive symptoms and MA among Thai youth living in rural areas using a complex sampling strategy consisting of multistage, community-clustered and systematic random sampling. Such a study sampling method was necessary given the ubiquity of MA trafficking and distribution in this region. Consistent with our hypothesis, high levels of depressive symptoms were positively associated with MA use.

We observed high rates of depressive symptoms in the entire sample and among MA users. In our study, approximately 12 % of individuals who reported ever using methamphetamine had high levels of depressive symptoms. The prevalence of depression among MA users is comparable with the estimates reported among psychostimulant users in the U.S. [23]. There are several possible explanations for observing this association. First, this population could possibly be suffering psychological sequelae of chronic MA use. Second, adolescents and young adults could initiate MA use as an attempt to self-medicate an existing depression [24]. The historical, social, economic and political context existing within Thailand is such that there is a change to a more competitive, global market-driven economy from a rural, agrarian lifestyle. This new social and political environment is replacing traditional family values, and these changes may initiate the onset of stress and lead to the development of depression among youth who perceive they are responsible for competing in the global economy [4]. Moreover, in a region where the drug market renders MA to be widespread, accessible and inexpensive, economic stressors may lead to the onset of depression and subsequently lead to MA use by adolescents and young adults to self-medicate [25].

The relationship between MA and depression could potentially be bidirectional. Symptoms indicative of depression could lead to initiation and continuation of MA use. Consequently, affective and motivational dysfunction related to depression can occur as a result of chronic use and withdrawal, which could possibly contribute to the continuation of MA use for the maintenance of depressive symptoms. This maintenance of depressive symptoms is prolonged due to sustained use of drugs in greater amounts, more frequent use or injecting in order to relieve depressive symptoms resulting from chronic use [26]. One longitudinal epidemiological study attempted to address the nature of this relationship by assessing the temporality of MA use and depressive symptoms among MA users in Thailand. This study concluded that depressive symptoms were a consequence of MA use [12].

Of note is the observed independent crude and adjusted association between gender and high levels of depressive symptoms. Of the participants that endorsed high levels of depressive symptoms, 6 % were male compared to 12 % of females. On the other hand, approximately 33 % of males reported recent MA use compared to 23 % of females and explains the lack of effect modification by gender. We had hypothesized a priori that gender would be a confounder since previous literature has documented higher levels of depressive symptoms in females, whether induced by substance use or independent of substance use, and has documented that males are more likely to use illicit substances than females. Other covariates were added to the multivariate model if they met the aforementioned criteria for being included, but had little effect on the estimate describing MA use and high levels of depressive symptoms.

Treatment of depressive symptoms presenting with substance use and dependence of MA has been problematic. Several randomized controlled trials conducted to evaluate the efficacy of various antidepressants in relieving depressive symptoms and achieving cessation have found treatment to be inconsistently efficacious among individuals who were dependent on or abused MA or cocaine [27–30]. All of these trials have been conducted in urban areas of the United States, and most involve small sample sizes. An additional review of treatment options specific to MA users presenting with depression reveals a large gap in knowledge of effective, sustainable treatment options whether treatment options are psychological approaches, pharmacological approaches or both [31].

There are some limitations inherent in this study that must be considered. First, the study was cross-sectional. A temporal relationship between MA use and high levels of depressive symptoms cannot be established. Second, there could be underreporting of stigmatized behaviors and stigmatization of MA use by Thai communities remains a concern among youth [3]. Misclassification could result from self-reports, but our interviews were administered using Audio computer-assisted self-interview (ACASI) software where previous studies have shown little effect on internal validity in similar populations [32]. Another limitation that should be considered is that the parent study was not implemented solely to assess our hypothesis. Therefore, numerous possible other (unmeasured) confounders such as other psychiatric disorders were not included in the survey. However, such disorders would be rare in this population. Different time frames were used to measure recent MA use, polydrug use and alcohol consumption. We did not include measures of illicit drug polydrug or MA use within the past 30 days in our baseline survey. However, inferences about the independent association between high levels of depressive symptoms and recent MA use within the past 3 months were similar in our univariate and multivariate models when recent polydrug and alcohol use were incorporated into the model and the association supports previous findings. Last, the Thai cutoff of  ≥ 22 was based on research among a limited sample size and included only Thai adolescent males.

This study presents strengths relevant for drawing public health implications. The large sample size of the study population, the probabilistic sampling methodology, and the extensive coverage of several communities within sub-districts are strong characteristics of this study. Relatively little research has been conducted in Thailand that quantifies the prevalence of high levels of depressive symptoms among adolescents and young adults who use MA, either in clinical samples or the general population. Little mental health or substance abuse research, in general, has been conducted in Thailand, but recent efforts to address these knowledge gaps are leading to a burgeoning field of substance use and mental health research in Thailand, and this study contributes to that research base. Most notably, an assessment of Thailand’s mental health care services by the Thai Ministry of Health and the World Health Organization specified that only 1 % of health-related research published in peer reviewed journals in Thailand is mental health research [13]. To our knowledge, no assessments of the prevalence and associations between high levels of depressive symptoms and MA use have been conducted in rural Chiang Mai province, the epicenter of an existing MA epidemic. There is a known association between MA use and depression, but few resources for the treatment and prevention of depression and substance use exist in this rural region. These assessments of individual-level risk factors that are associated with high levels of depressive symptoms provide information that could be used to target prevention efforts among Thai youth who use MA in this region.

Additional longitudinal and qualitative studies should be conducted to examine how other individual-level factors and multiple-level factors (i.e. physical environment, social context, substance use, abuse and dependence) predict the onset of depressive symptoms among Thai youth to better understand the individual-level and structural factors driving the onset of depression. Longitudinal studies should also be designed to assess if depressive symptoms precede or are a consequence of substance use.

Generally, this study could be used as the basis to begin the evaluation of the effectiveness of treatment and prevention interventions for depressive symptoms associated with MA use in Thailand as well as other low- and middle-income countries. The positive association seen in our study suggests that accessible and culturally acceptable mental health care services should be integrated with drug treatment programs among drug-dependent youth. The results also indicated that policies aimed at maximizing resources in rural Thailand to deliver more humane and evidence-based approaches to care and treatment of depressive symptoms among MA users could contribute to the prevention of MA use and the reduction of depressive symptoms among Thai youth.

## Conclusion

Associations between MA use and high levels of depressive symptoms were observed. Given the ubiquity of MA use among adolescents and young adults in this region of Thailand, resources should target this population for the provision of culturally acceptable mental health care services for MA-dependent Thai youth.

## Abbreviation

MA: methamphetamine
